# A Study of Inverted-Type Perovskite Solar Cells with Various Composition Ratios of (FAPbI_3_)_1−*x*_(MAPbBr_3_)*_x_*

**DOI:** 10.3390/nano6100183

**Published:** 2016-10-13

**Authors:** Lung-Chien Chen, Zong-Liang Tseng, Jun-Kai Huang

**Affiliations:** Department of Electro-Optical Engineering, National Taipei University of Technology, No. 1, Sec. 3, Chung-Hsiao E. Rd., Taipei 10608, Taiwan; tw78787788@yahoo.com.tw (Z.-L.T.); t103658032@ntut.org.tw (J.-K.H.)

**Keywords:** MAPbBr_3_, FAPbI_3_, solar cells, perovskite

## Abstract

This work presents mixed (FAPbI_3_)_1−*x*_(MAPbBr_3_)*_x_* perovskite films with various composition ratios, *x* (*x* = 0–1), which are formed using the spin coating method. The structural, optical, and electronic behaviors of the mixed (FAPbI_3_)_1−*x*_(MAPbBr_3_)*_x_* perovskite films are discussed. A device with structure glass/indium tin oxide (ITO)/poly(3,4-ethylenedioxythiophene) polystyrene sulfonate (PEDOT:PSS)/mixed perovskite/C_60_/BCP/Ag was fabricated. The mixed perovskite film was an active light-harvesting layer. PEDOT:PSS was a hole transporting layer between the ITO and perovskite. Both C_60_ and bathocuproine (BCP) were electron transporting layers. MAPbBr_3_ was added to FAPbI_3_ with a composition ratio of *x* = 0.2, stabilizing the perovskite phase, which exhibited a uniform and dense morphology. The optimal device exhibited band matching with C_60_, resulting in a low series resistance (R_sh_) and a high fill factor (FF). Therefore, the device with composition (FAPbI_3_)_1−*x*_(MAPbBr_3_)*_x_* and *x* = 0.2 exhibited outstanding performance.

## 1. Introduction

Organometal halide perovskite solar cells have been intensively investigated owing to their high power conversion efficiency and fabrication in solution at low temperatures. Perovskite solar cells with an efficiency of over 20% have been fabricated [1,2,3,4]. The excellent performance of organometal halide perovskite solar cells has two causes: a small bandgap and a large exciton diffusion length. The low absorption bandgap (E_g_ of CH_3_NH_3_PbI_3_ (MAPbI_3_) ~1.5 eV) of organometal halide perovskite can harvest most wavelengths of incident sunlight. The long exciton diffusion length increases the thickness of active light-harvesting layers and ensures efficient carrier generation [5,6]. Moreover, the conventional structure of perovskite solar cells use TiO_2_ for electron transport, but the TiO_2_ needs to be processed using high temperatures (500–600 °C), and many applications are limited. Inverted-type perovskite solar cells have been developed for low-temperature process and low hysteresis [7].

Interestingly, the compositional engineering of perovskite materials has been extensively utilized to adjust their bandgap and structural properties for use in efficient perovskite solar cells. CH_3_NH_3_PbI_3−*x*_Br*_x_* (*x* = 0.1–0.15), as an absorbing layer, has been reported to improve the open voltage of photovoltaic devices [8]. CH_3_NH_3_PbI_3−*x*_Cl*_x_* has been used to increase the exciton diffusion length to improve device performance [9,10,11,12]. HC(NH_2_)_2_PbI_3_ (FAPbI_3_) [13,14,15,16] can reduce the optical bandgap (Eg ~1.48 eV), with an absorption edge of 840 nm, allowing photons to be absorbed over a broader solar spectrum. Accordingly, FAPbI_3_ absorbs more light than MAPbI_3_. Another advantage of FAPbI_3_ is its thermal stability [17]. It can be processed at a higher temperature than can MAPbI_3_. The typical annealing process temperature is approximately 130–170 °C. In particular, Jeon et al. explained that (FAPbI_3_)_1−*x*_(MAPbBr_3_)*_x_* can provide a greater balance between electron transport and the hole transport in cells, enabling highly efficient perovskite solar cells to be formed using a regular TiO_2_ mesoscopic structure (>20%) [4]. However, the origin of their favorable performances and their fabrication process are not yet fully understood.

In this work, solution-processed (FAPbI_3_)_1−*x*_(MAPbBr_3_)*_x_* perovskites were prepared on poly(3,4-ethylenedioxythiophene) polystyrene sulfonate (PEDOT:PSS)-coated indium tin oxide (ITO) substrates for use in inverted perovskite solar cells. The optical, structural, and surface properties of the (FAPbI_3_)_1−*x*_(MAPbBr_3_)*_x_* perovskite films were studied as functions of the composition ratio (*x* = 0–1). The dependence of cell performance and the properties of the perovskite films is discussed.

## 2. Materials and Methods

**Figure 1:**
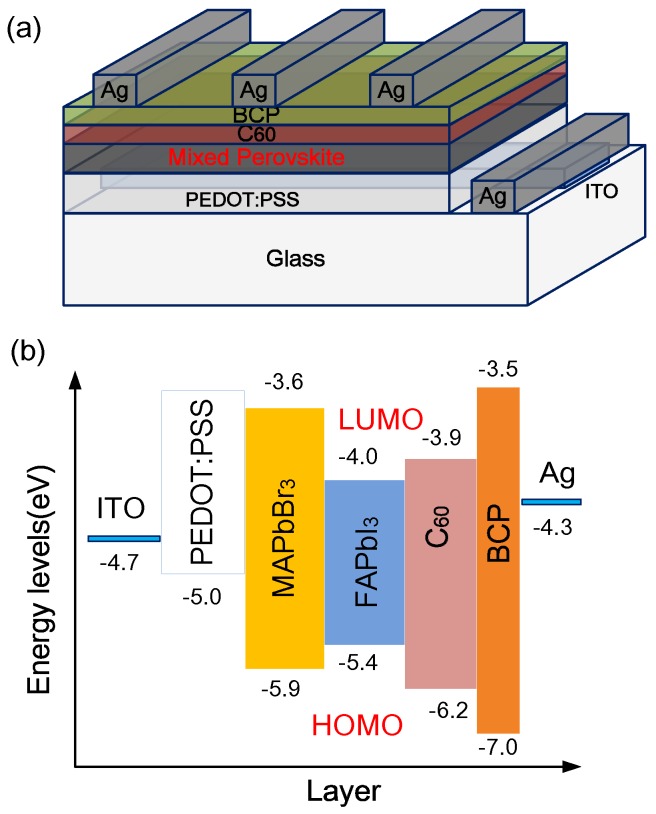
(**a**) Complete structure and (**b**) corresponding energy band diagram of the structure.

In this work, a PEDOT:PSS (AI 4083) was spin-coated on a pre-cleaned ITO substrate at 5000 rpm for 30 s. Thereafter, the film was annealed at 120 °C for 10 min. The perovskite layer was deposited as in our previous investigation [18]. HC(NH_2_)_2_I (FAI), PbI_2_, CH_3_NH_3_Br (MABr), and PbBr_2_ were dissolved in 1 mL of cosolvent, which comprised dimethyl sulfoxide (DMSO) and γ-butyrolactone (GBL) (volume ratio = 1:1), to form perovskite precursor solutions. The mole ratios of FAPbI_3_ to MAPbBr_3_ in the mixed perovskite varied from 0 to 1. The concentrations of each precursor were 1.2 M. For example, 0.96 mmol of FAPbI_3_ and 0.24 mmol of MAPbBr_3_ (i.e., 165 mg of FAI, 27 mg of MABr, 443 mg of PbI_2_, and 88 mg of PbBr_2_) were dissolved in the mixing solvent (1 mL) as a (FAPbI_3_)_0.8_(MAPbBr_3_)_0.2_ precursor solution. The perovskite precursor solutions were then coated onto the PEDOT:PSS/ITO substrate in two consecutive spin-coating steps, at 1000 rpm and 5000 rpm for 10 s and 20 s, respectively, in a glove box that was filled with highly pure nitrogen (>99.999%). The wet spinning film was quenched by dropping 50 μL of anhydrous toluene at 17 s. After spin coating, the film was annealed at 100 °C for 10 min. Subsequently, C_60_, Bathocuproine (BCP), and a silver (Ag) electrode were deposited with thicknesses of 50, 5, and 100 nm, respectively, using a thermal evaporator. The sample was covered with a shadow mask to define an active area of 0.5 cm × 0.2 cm during C_60_/BCP/Ag deposition. Figure 1a schematically depicts the complete structure.

### Material and Device Measurement

The crystalline microstructures of the films were determined using a PAN analytical X’Pert Pro DY2840 X-ray diffractometer (PANalytical, Naerum, Denmark) with CuKα radiation (λ = 0.1541 nm). A field-emission scanning electron microscope (GeminiSEM, ZEISS, Oberkochen, Germany) was used to observe the surface morphology of the cells. Photoluminescence (PL) and absorption spectra were measured using a fluorescence spectrophotometer (Hitachi F-7000) and a UV/VIS/NIR spectrophotometer (Hitachi U-4100 spectrometers) (Hitachi High-Technologies Co., Tokyo, Japan), respectively. Current density–voltage (J-V) characteristics were measured using a Keithley 2420 programmable source meter (Keithley, Cleveland, OH, USA) under illumination by a 1000 W xenon lamp. The forward scan rate was 0.1 V/s.

## 3. Results and Discussion

Mixed perovskite film can be flexibly modified by changing the concentration ratio of the precursors [4,19]. The lowest unoccupied molecular orbitals (LUMOs) of FAPbI_3_, MAPbBr_3_, and C_60_ are −4.0, −3.6, and −3.9 eV, respectively [20,21]. The bandgap of the mixed (FAPbI_3_)_1−*x*_(MAPbBr_3_)*_x_* film is between that of the FAPbI_3_ film and that of the MAPbBr_3_ film, from −4.0 to −3.6. To optimize the band matching with C_60_, the composition ratio x of FAPbI_3_ to MAPbBr_3_ in the mixed (FAPbI_3_)_1−*x*_(MAPbBr_3_)*_x_* perovskite is approximately 0.2 because the LUMO of the (FAPbI_3_)_0.__75_(MAPbBr_3_)_0.2__5_ was determined by the interpolation to be −3.9, as shown in Figure 1b. Therefore, the device with the mixed (FAPbI_3_)_0.75_(MAPbBr_3_)_0.25_ perovskite film should have the lowest series resistance (R_sh_).

**Figure 2:**
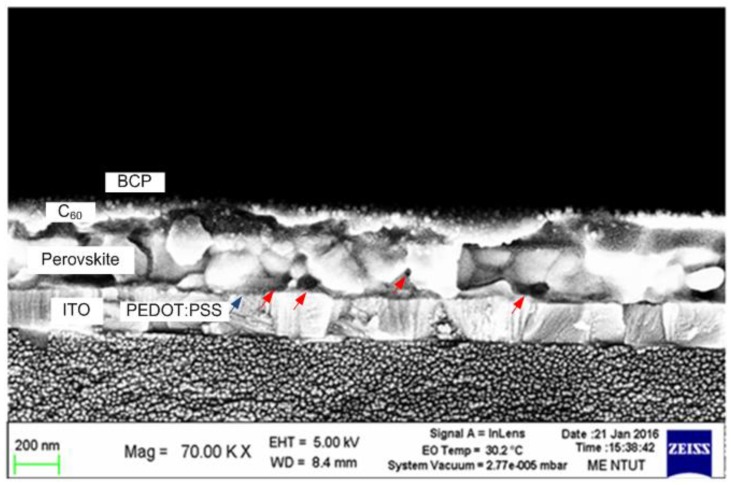
Field emission scanning electron microscope (FESEM) cross-sectional image of device structure.

Figure 2 displays a high-resolution image of the cross-section of the obtained perovskite solar cell configuration, which clearly shows the presence of the layers ITO (200 nm), PEDOT:PSS (~50 nm), perovskite (~250 nm), C_60_ (~60 nm), and BCP (~10 nm). The grain size of the perovskite is approximately 200 nm, as presented in Figure 2. Numerous voids (indicated by red arrows) between grain boundaries were observed. These are characteristic of mixed (FAPbI_3_)_1−*x*_(MAPbBr_3_)*_x_* perovskite films and may be attributed to the supersaturation nucleation and dynamic growth mechanism [22].

**Figure 3:**
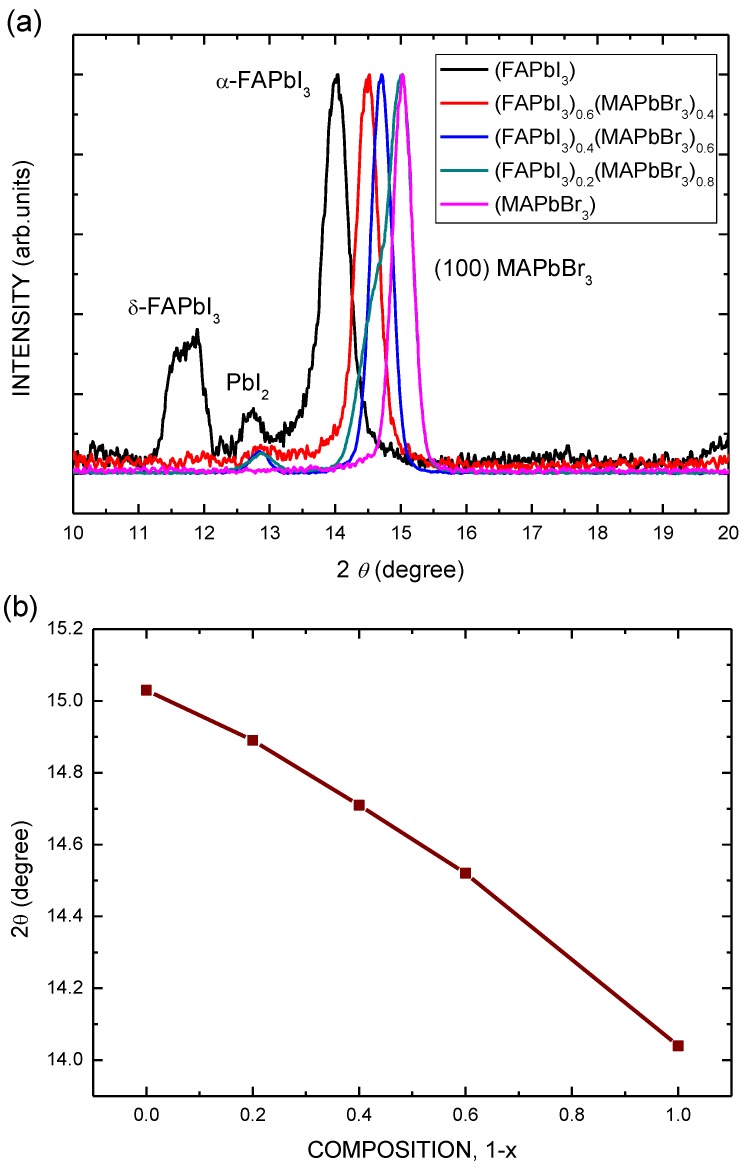
(**a**) X-ray diffraction (XRD) patterns of (FAPbI_3_)_1−*x*_(MAPbBr_3_)*_x_* perovskite films with various compositions; (**b**) Relationship between degree of diffraction and composition of (FAPbI_3_)_1−*x*_(MAPbBr_3_)*_x_*.

Figure 3a shows the X-ray diffraction (XRD) patterns of (FAPbI_3_)_1−*x*_(MAPbBr_3_)*_x_* perovskite films after thermal annealing at various temperatures. The spectrum of the MAPbBr_3_ film includes three main diffraction peaks at 14.04°, which correspond to the δ-FAPbI_3_, PbI_2_, and α-FAPbI_3_ phases, respectively. As the value of *x* in the (FAPbI_3_)_1−*x*_(MAPbBr_3_)*_x_* perovskite films increases, the position of the α-FAPbI_3_ phase peak shifts considerably to a high degree of diffraction, and the δ-FAPbI3 and PbI_2_ phase peaks disappear. The coexistence of the two FAPbI_3_ and PbI_2_ phases can be observed in the (FAPbI_3_)_1−*x*_(MAPbBr_3_)*_x_* perovskite layers with FAPbI_3_. The spectrum of the MAPbBr_3_ film includes one diffraction peak at 15.03°, which corresponds to the (100) phase. As presented in Figure 3b, the peak position increases almost linearly with *x*, revealing that the crystalline FAPbI_3_ and MAPbBr_3_ are homogeneous.

**Figure 4:**
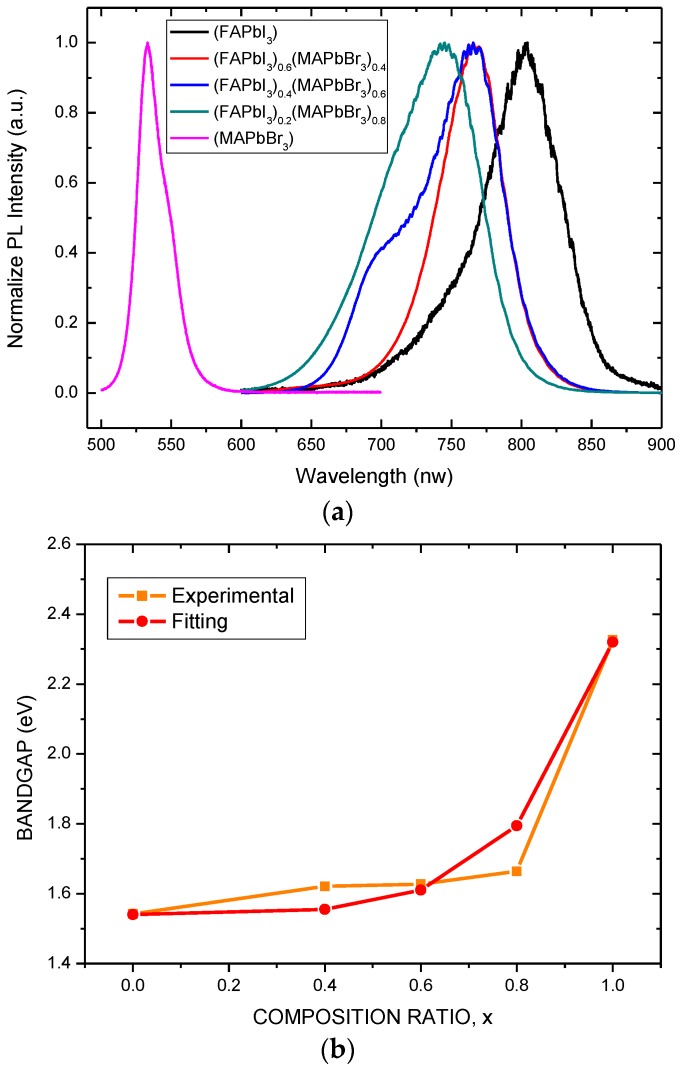
(**a**) Photoluminescence (PL) spectra of mixed (FAPbI_3_)_1−*x*_(MAPbBr_3_)*_x_* perovskite films with various values of *x*. (**b**) Bandgap of mixed (FAPbI_3_)_1−*x*_(MAPbBr_3_)*_x_* perovskite films, estimated from PL spectra.

Figure 4a presents the room-temperature PL spectra of (FAPbI_3_)_1−*x*_(MAPbBr_3_)*_x_* films with various composition ratios that were deposited on glass substrates. The PL peak shifts nonlinearly from 804 to 533 nm as the composition ratio *x* is increased from 0 to 1, as displayed in Figure 4a. The bandgaps of MAPbBr_3_ and FAPbI_3_ are approximately 2.3 and 1.5 eV, respectively, and correspond to wavelengths of around 540 and 820 nm, respectively. Therefore, bandgap values and PL results match. The bandgap of the mixed (FAPbI_3_)_1−*x*_(MAPbBr_3_)*_x_* perovskite films is calculated from the PL spectra, as shown in Figure 4b. The bandgap over the entire range of the (FAPbI_3_)_1−*x*_(MAPbBr_3_)*_x_* perovskite films can be estimated from the PL spectra. Fitting the PL spectra at room temperature yields the following expression for the bandgap, Eg:

Eg(*x*) = 1.5 + 0.2*x*^3^ + 0.58*x*^6^. (1)

The expression is a sixth-order polynomial, rather than the traditional second-order polynomial for compound semiconductors, revealing that the bandgap of the mixed (FAPbI_3_)_1−*x*_(MAPbBr_3_)*_x_* perovskite films is extremely sensitive to the composition of the mixed (FAPbI_3_)_1−*x*_(MAPbBr_3_)*_x_* perovskite films when the concentration of the FAPbI_3_ is low.

**Figure 5:**
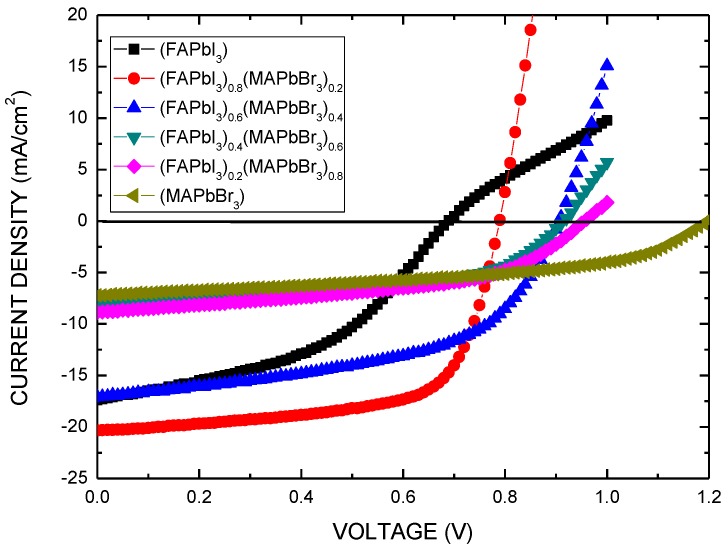
J-V curves of perovskite solar cell (Ag/BCP/C_60_/(FAPbI_3_)_1−*x*_(MAPbBr_3_)*_x_*/PEDOT:PSS/ITO) obtained under standard 1 sun air mass (AM) 1.5 simulated solar irradiation.

Figure 5 plots the current density as a function of the voltage (J-V) of solar cells that are based on (FAPbI_3_)_1−*x*_(MAPbBr_3_)*_x_* films with various composition ratios. Table 1 presents the power conversion efficiency (Eff), short-circuit current density (J_sc_), open-circuit voltage (V_oc_), and fill factor (FF) of the (FAPbI_3_)_1−*x*_(MAPbBr_3_)*_x_* solar cells. The bandgap of the (FAPbI_3_)_1−*x*_(MAPbBr_3_)*_x_* film is reduced as the proportion of (MAPbBr_3_) in the (FAPbI_3_)_1−*x*_(MAPbBr_3_)*_x_* films increases. The power conversion efficiency increases with *x* in the (FAPbI_3_)_1−*x*_(MAPbBr_3_)*_x_* films because J_sc_ increases with the strength of absorption and the amount of α-FAPbI_3_ formed. However, the power conversion efficiency decreases as more MAPbBr_3_ is formed owing to a reduction in the photocurrent and series resistance (R_sh_). The optimal device, with a (FAPbI_3_)_0.8_(MAPbBr_3_)_0.2_ perovskite film, exhibited outstanding performance, where J_sc_ = 20.6 mA/cm^2^, V_oc_ = 0.88 V, FF = 65.9%, and Eff = 12.0%. MAPbBr_3_ was added to FAPbI_3_ where *x* = 0.2 to stabilize the perovskite phase with a uniform and dense morphology [4]. Therefore, the device with the (FAPbI_3_)_0.8_(MAPbBr_3_)_0.2_ perovskite film exhibited the lowest series resistance (R_sh_), the best FF, and therefore the best performance.

The bandgap of the (FAPbI_3_)_1−*x*_(MAPbBr_3_)*_x_* film is reduced as the amount of MAPbBr_3_ in the (FAPbI_3_)_1−*x*_(MAPbBr_3_)*_x_* film increases. Therefore, by increasing the composition ratio *x*, the (FAPbI_3_)_1−*x*_(MAPbBr_3_)*_x_* film increases the LUMO of the (FAPbI_3_)_1−*x*_(MAPbBr_3_)*_x_* film to match that of the C_60_ layer and increases the energy barrier to the transportation of electrons, resulting in a low FF. The value of V_oc_ is positively correlated with the difference between the highest occupied molecular orbital (HOMO) of the (FAPbI_3_)_1−*x*_(MAPbBr_3_)*_x_* film and the LUMO of the C_60_ layer (Figure 1b) [23]. Therefore, V_oc_ is determined by the increase in the HOMO level of the (MAPbBr_3_)_*x*_(FAPbI_3_)_1−*x*_ film. The bandgap of the (FAPbI_3_)_1−*x*_(MAPbBr_3_)*_x_* film increases with *x*, increasing V_oc_. Additionally, the LUMO level of the (MAPbBr_3_)*_x_*(FAPbI_3_)_1−*x*_ film is lower than that of C_60_, so an energy barrier is formed, lowering FF. Additionally, the photo-generated current density declines as the proportion of MAPbBr_3_ in the (FAPbI_3_)_1−*x*_(MAPbBr_3_)*_x_* films increases because the absorption range is reduced.

## 4. Conclusions

In summary, this work presents mixed (FAPbI_3_)_1-x_(MAPbBr_3_)_x_ perovskite films with various composition ratios (*x* = 0 to 1), formed using the spin coating method. The bandgap over the entire range of the (FAPbI_3_)_1−*x*_(MAPbBr_3_)*_x_* perovskite films can be estimated from the PL spectra. Fitting the PL spectra yields a sixth-order polynomial for the bandgap of the mixed (FAPbI_3_)_1−*x*_(MAPbBr_3_)*_x_* perovskite films. This result reveals that the bandgap of the mixed (FAPbI_3_)_1−*x*_(MAPbBr_3_)*_x_* perovskite films is extremely sensitive to the composition of the mixed (FAPbI_3_)_1−*x*_(MAPbBr_3_)*_x_* perovskite films when the concentration of the FAPbI_3_ is low. The optimal device uses (FAPbI_3_)_1−*x*_(MAPbBr_3_)*_x_* with *x* = 0.2 and exhibited outstanding performance, where short-circuit current density J_sc_ = 20.6 mA/cm^2^, open-circuit voltage V_oc_ = 0.88 V, fill factor FF = 65.9%, and power conversion efficiency Eff = 12.0%, perhaps because the addition of MAPbBr_3_ to FAPbI_3_ where *x* = 0.2 stabilized the perovskite phase with a uniform and dense morphology. The optimum device exhibits band matching with C_60_, resulting in a low series resistance (R_sh_) and high FF.

## Figures and Tables

**Table 1:** Parameters of solar cells based on perovskite (FAPbI_3_)_1−*x*_(MAPbBr_3_)*_x_* film with various composition ratios.

(FAPbI_3_)_1−*x*_(MAPbBr_3_)*_x_*	V_oc_ (V)	J_sc_ (mA/cm^2^)	FF (%)	Eff (%)	R_sh_ (Ω)
(FAPbI_3_)	0.60	17.3	44.0	5.68	20.3
(FAPbI_3_)_0.8_(MAPbBr_3_)_0.2_	0.88	20.6	65.9	12.0	4.6
(FAPbI_3_)_0.6_(MAPbBr_3_)_0.4_	0.90	17.63	52.9	9.41	8.5
(FAPbI_3_)_0.4_(MAPbBr_3_)_0.6_	0.90	11.01	51.4	5.51	19.8
(FAPbI_3_)_0.2_(MAPbBr_3_)_0.8_	0.95	8.86	49.4	4.18	21.2
(MAPbBr_3_)_1_	1.2	7.23	47.7	4.19	30.6
